# Effect of weekly versus daily primaquine on *Plasmodium vivax* malaria recurrences: A real-life cohort study

**DOI:** 10.1590/0037-8682-0738-2021

**Published:** 2022-04-29

**Authors:** Jose Diego Brito-Sousa, Jeffe Phanor, Patricia Carvalho da Silva Balieiro, Alexandre Vilhena Silva-Neto, Jady Shayenne Mota Cordeiro, Sheila Vitor-Silva, Maxwell Mendes, Vanderson Souza Sampaio, Gisely Cardoso de Melo, Marcus Lacerda, Wuelton Monteiro

**Affiliations:** 1 Fundação de Medicina Tropical Doutor Heitor Vieira Dourado, Instituto de Pesquisa Clínica Carlos Borborema, Manaus, AM, Brasil.; 2 Universidade do Estado do Amazonas, Escola Superior de Ciências da Saúde, Manaus, AM, Brasil.; 3 Universidade Federal do Amazonas, Escola de Enfermagem de Manaus, Manaus, AM, Brasil.; 4 >Fundação de Vigilância em Saúde do Amazonas Dra. Rosemary Costa Pinto, Manaus, AM, Brasil.; 5 Fundação Oswaldo Cruz, Instituto Leônidas & Maria Deane, Manaus, AM, Brasil.

**Keywords:** Weekly primaquine, Recurrences, Brazilian Amazon, Elimination, G6PD deficiency

## Abstract

**Background:**

Although primaquine (PQ) is indicated for G6PD-deficient patients, data on weekly PQ use in Brazil are limited.

**Methods:**

We aimed to investigate malaria recurrences among participants receiving daily and weekly PQ treatments in a real-life setting of two municipalities in the Amazon between 2019 and 2020.

**Results:**

Patients receiving weekly PQ treatment had a lower risk of recurrence than those receiving daily PQ treatment (risk ratio: 0.62, 95% confidence interval: 0.41-0.94), using a model adjusted for study site.

**Conclusions:**

Weekly PQ use did not increase the risk of malaria recurrence. Further studies with larger populations are warranted.

A great challenge to the elimination of *Plasmodium vivax* malaria has been imposed by glucose 6-phosphate dehydrogenase deficiency (G6PDd) in malaria-endemic areas^1^. The use of 8-aminoquinolines, such as primaquine (PQ) and tafenoquine (TQ), which are required for the radical cure of *P. vivax* malaria, is associated with severe and often life-threatening hemolysis, even in patients with enzymatic variants that are considered to be mild to moderate^2^ ^,^ ^3^. A weekly PQ treatment (0.75 mg/kg/week for 8 weeks) has been suggested for G6PD-deficient patients, provided that close monitoring of severe hemolysis is possible in areas where more severe enzyme variants prevail^4^. This regimen is usually tolerable, with the decrease in hemoglobin level being transient within the first weeks, which allows patients to recover after each dose^5^ ^-^ ^7^. 

Although safer for G6PDd patients than the standard treatment (daily PQ), a recent systematic review could not show whether a weekly PQ treatment is as effective as the 14-day PQ treatment in preventing recurrences of *P. vivax* malaria^8^. Additionally, data related to its efficacy in a real-world setting are not available. Thus, this study aimed to compare the incidence of malaria recurrence among patients using daily the standard PQ treatment (0.5 mg/kg/day for 7 days) and those using weekly PQ treatment in real-life situations in two municipalities in the Brazilian Amazon.

This study was part of the Safeprim mixed-method study, which evaluated the implementation of G6PDd screening tools in malaria treatment units (MTUs) of two municipalities in the Brazilian Amazon^9^ ^,^ ^10^. The CareStart^TM^ qualitative test (Acess Bio, New Jersey, USA) was implemented in Rio Preto da Eva, Amazonas state (February 2019 to early January 2020), while the Standard^TM^ G6PD quantitative test (SD Biosensor, Korea) was implemented in both Mâncio Lima, Acre state (January to December 2020) and Rio Preto da Eva (January to August 2020). At the MTUs, when the thick blood smear tested positive for *P. vivax*, the PQ regimen was chosen by the healthcare workers based on the results of the G6PD test: chloroquine plus daily (0.5 mg/kg/day for 7 days) or weekly (0.75 mg/kg/week for 8 weeks) PQ was administered to patients with normal and deficient G6PD status, respectively, in accordance with the Brazilian Ministry of Health guidelines^11^. A deficient result was obtained if a patient presented no color or a very faint color change on CareStart reading window and if the Standard biosensor screen showed a G6PD level of <4.0 IU/gHb.

All patients’ data were collected and transferred to the national malaria reporting system (SIVEP-Malaria) by the healthcare teams in each municipality. During the implementation of CareStart^TM^, data were collected using REDCap forms in which all mandatory fields were automatically transferred to SIVEP-Malaria, which at that time did not include information on G6PD deficiency. For the Standard^TM^ test, data were entered directly into the SIVEP-Malaria database, which was later adapted to receive G6PDd information as well. 

Descriptive statistics were used to analyze the demographic data. To assess the recurrence rates between patients with normal G6PD levels and G6PD deficiency, patients were followed up for 180 days after the end of treatment using a model adjusted by study site as the grouping variable. Probabilistic data linkage was used for the deduplication of successive cases from the same patient, as described elsewhere^12^. A Cox proportional hazards model was used in the analysis of time-to-event data adjusted for the study site. All analyses were performed using the R software v.4.1.0, R Studio v.1.4.1717, and Stata v17. This study was approved by the Ethics Review Board of *Fundação de Medicina Tropical Dr. Heitor Vieira Dourado*, Manaus, Brazil (CAAE: 92012818.1.0000.0005).

The results of 122 G6PD-deficient and 1,322 normal G6PD patients were reported between February 2019 and December 2020 in both municipalities. Table 1 summarizes the patients’ demographic characteristics.

**TABLE 1:** Demographic characteristics of the study participants.

	PQ Treatment		
	Total	Weekly	Daily
**Variable**	*n*=1,444	*n*=122	*n*=1,322
**Municipality (*n*=1,444; 100%)**			
Rio Preto da Eva, AM	450 (31.2%)	83 (68.0%)	367 (27.8%)
Mâncio Lima, AC	994 (68.8%)	39 (32.0%)	955 (72.2%)
**Age (SD)**	27.3 (18.0)	30.2 (19.8)	27.0 (17.9)
**Gender (female) (*n*=1,444; 100%)**	558 (38.6%)	53 (43.4%)	505 (38.2%)
**School education (*n*=1,288; 89.20%)**			
Illiterate	23 (1.8%)	8 (7.0%)	15 (1.3%)
Incomplete elementary school	928 (72.0%)	76 (66.7%)	852 (72.6%)
Complete primary education	106 (8.2%)	10 (8.8%)	96 (8.2%)
Incomplete high school	77 (6.0%)	8 (7.0%)	69 (5.9%)
Complete high school	127 (9.9%)	12 (10.5%)	115 (9.8%)
Incomplete higher education	9 (0.7%)	0 (0.0%)	9 (0.8%)
Complete higher education	18 (1.4%)	0 (0.0%)	18 (1.5%)
**Zone of residence (*n*=1,299; 89.96%)**			
Rural	1,165 (89.7%)	79 (87.8%)	1,086 (89.8%)
Urban	134 (10.3%)	11 (12.2%)	123 (10.2%)
**Parasitemia (mm** ^3^ **) at baseline (*n*=1,253; 86.77%)**			
<199	242 (19.3%)	16 (14.3%)	226 (19.8%)
200-300	311 (24.8%)	21 (18.8%)	290 (25.4%)
301-5,000	235 (18.8%)	15 (13.3%)	220 (19.3%)
501-10,000	346 (27.6%)	33 (29.5%)	313 (27.4%)
10,001-100,000	119 (9.5%)	27 (24.1%)	92 (8.1%)
**At least one recurrence by day 180 *(n*=1,444; 100%)**	285 (19.7%)	20 (16.4%)	265 (20.0%)
**Number of recurrences in 180 days (*n*=285;19.74%)**			
1	223 (78.2%)	15 (75.0%)	208 (78.5%)
2	42 (14.7%)	3 (15.0%)	39 (14.7%)
3	17 (6.0%)	2 (10.0%)	15 (5.7%)
4	3 (1.1%)	0 (0.0%)	3 (1.1%)

**G6PD:** glucose 6-phopsphate dehydrogenase; **AM:** Amazonas; **AC:** Acre; **SD:** standard deviation.

When comparing the recurrence rates between patients with normal G6PD and those with G6PD deficiency and considering them as a proxy for daily versus weekly PQ treatment, 20 of 122 patients (16.4%) who were taking PQ weekly had recurrence within 180 days compared with 265 of 1,322 patients (20.0%) who were taking PQ daily (risk ratio: 0.62, 95% confidence interval: 0.41-0.94, using a model adjusted for the study site. Figure 1 shows the time to the first recurrence between the groups.

**FIGURE 1:**
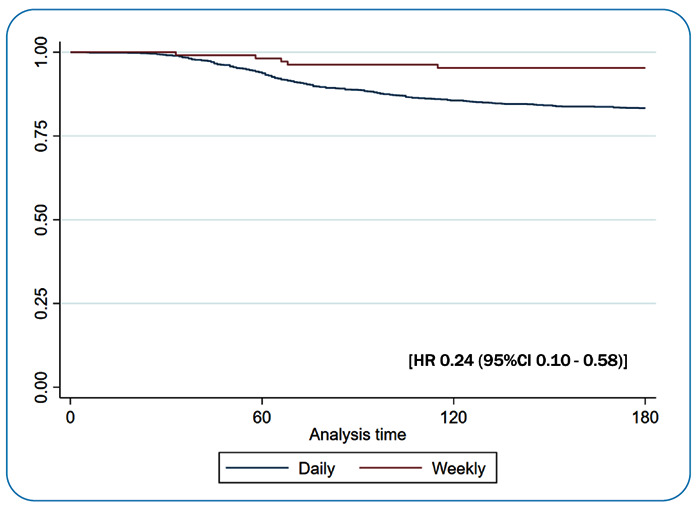
Time to first recurrence between groups using weekly and daily PQ regimens within 180 days. Cox-proportional hazard model was used adjusted by study site. **HR:** hazard ratio; **CI:** confidence interval.

The recurrence rates were lower in patients receiving weekly (16.4%) versus daily (20%) PQ. The weekly use of PQ was recently added to the updated Brazilian Ministry of Health guidelines^11^; however, routine screening for G6PDd is not currently performed prior to the treatment of malaria. The safer option (weekly PQ treatment) allows clinical recovery between doses, since hemoglobin values usually return to baseline within the first 2-3 weeks^7^. 

Non-adherence to standard treatment in the Brazilian Amazon can contribute to the recurrence of malaria^13^ ^,^ ^14^. Patients with G6PDd can be prone to recurrence when the test is not performed, possibly due to treatment discontinuation owing to the patients’ fear of undergoing hemolysis when using daily PQ treatment [Nascimento et al., personal communication]. With the recent availability of single-dose tafenoquine for the radical cure of *P. vivax*, adherence among patients with normal G6PD levels may be ensured; however, this depends on the availability of suitable and reliable G6PDd screening tools at the point of care^15^. Nevertheless, adherence to the longer weekly course of PQ in G6PDd patients still needs to be addressed, and further improvements should be discussed by policymakers and local healthcare staff.

This study has several limitations. This was an exploratory analysis; hence, the main study was not specifically designed to perform this comparison. Treatment supervision cannot be ensured for both daily and weekly treatments, as this mostly depends on the availability of personnel in both municipalities. The entry of data into the national malaria database was dependent solely on the teams in each municipality. However, as a real-life evidence study, we aimed to assess the implementation pragmatically, including all aspects. Even the use of a screening tool and patients already diagnosed with G6PD deficiency could influence the weekly treatment, thus causing it to be more supervised than the standard treatment. Although the study was conducted during the coronavirus disease 2019 (COVID-19) pandemic, the malaria diagnosis was not interrupted since it was provided at the basic unit level.

In conclusion, patients receiving weekly PQ treatment had a lower risk of recurrence than those receiving daily PQ treatment in these two municipalities. However, further studies are necessary to assess its efficacy in larger populations with different healthcare levels.
